# The prognostic value of inflammation-based scores in advanced hepatocellular carcinoma patients prior to treatment with sorafenib

**DOI:** 10.18632/oncotarget.21401

**Published:** 2017-09-30

**Authors:** Guillaume Conroy, Julia Salleron, Arthur Belle, Mouni Bensenane, Abdelbasset Nani, Ahmet Ayav, Didier Peiffert, Anthony Lopez, Cédric Baumann, Hélène Barraud, Jean-Pierre Bronowicki

**Affiliations:** ^1^ INSERM U954, Department of Hepato-gastroenterology, Lorraine University, Nancy University Hospital, Vandœuvre-lès-Nancy, France; ^2^ Department of Biostatistics, Lorraine Comprehensive Cancer Center, Vandœuvre-lès-Nancy, France; ^3^ Department of Digestive, Hepatobiliary and Endocrine Surgery, Lorraine University, Nancy University Hospital, Nancy, France; ^4^ Department of Radiotherapy, Lorraine University, Lorraine Comprehensive Cancer Center, Vandœuvre-lès-Nancy, France; ^5^ ESPRI-BioBase Unit, Platform of PARC, Nancy University Hospital, Vandœuvre-lès-Nancy, France

**Keywords:** HCC, inflammation-based scores, sorafenib, prognostic factor

## Abstract

**Background and Aims:**

The multikinase inhibitor sorafenib is the only currently approved drug for the indication of advanced hepatocellular carcinoma (HCC). It provides a limited gain in survival time but is frequently associated with adverse events. We currently lack simple prognostic factors in sorafenib-treated HCC patients. Various inflammation-based scores (IBSs) have been evaluated as predictors of tumor recurrence and survival in various malignancies (including HCC). The objective of the present study was to determine the prognostic value of IBSs for overall survival (OS) in advanced HCC patients prior to the initiation of sorafenib therapy.

**Methods:**

Patients with Barcelona Clinic Liver Cancer stage C HCC were enrolled retrospectively between October 2007 and September 2015. To identify prognostic factors for OS, bivariate and multivariate analysis were performed using a Cox proportional hazards regression model.

**Results:**

161 patients (87.0% males; median age: 67; median OS: 9.1 months) were enrolled. A multivariate analysis identified a body mass index <25kg/m^2^ (hazard ratio (HR)=1.55, p<0.017), macroscopic vascular invasion (HR=1.63, p< 0.001), an AST level >38 U/L (HR=2.65, p<0.001), Child Pugh B stage (HR=2.59, p<0.001) and a systemic immune-inflammation index (SII) ≥600 × 10^9^ (HR 1.72, p=0.002) as independent risk factors for OS in advanced HCC.

**Conclusion:**

IBSs (such as the SII) are novel, simple, low-cost prognostic indices in patients with advanced HCC. They may be of value in determining whether these patients may benefit from sorafenib therapy.

## INTRODUCTION

Hepatocellular carcinoma (HCC) is the sixth most common cancer and the second leading cause of cancer-related death in the world [1]. Early-stage HCC is poorly symptomatic, and so most cases are diagnosed at an advanced stage [2]. The oral multi-tyrosine kinase inhibitor sorafenib is the only currently authorized drug for Barcelona Clinic Liver Cancer (BCLC) stage C-HCC. Sorafenib treatment is associated with a limited gain in survival (around three months, giving a median overall survival (OS) time of 10.7 months in the SHARP trial [3, 4]). Advanced HCC is a heterogeneous disease; it includes symptomatic tumors and those with an invasive tumor pattern (i.e. macroscopic vascular invasion/extrahepatic spread) [5–7]; hence, reliable predictive factors for clinical outcomes in sorafenib-treated patients have yet to be identified [8]. Recent attention has been focused on cancer-associated inflammation - a key determinant of disease progression and survival in several solid tumors. It has been shown that scoring systems based on the systemic inflammatory response (referred to below as inflammation-based scores (IBSs)) are of prognostic value in patients with solid tumors [9–17]. In particular, various combinations of hematological parameters (including the neutrophil-lymphocyte ratio (NLR), the derived-NLR (dNLR), the lymphocyte-to-monocyte ratio (LMR), the platelet-lymphocyte ratio (PLR), the systemic-immune inflammation index (SII) and the prognostic nutritional index (PNI)) have been evaluated as predictors of recurrence and/or survival in HCC [18–27]. The objective of the present study was to investigate the prognostic value of pre-treatment IBSs for the OS of advanced HCC patients subsequently treated with sorafenib.

## RESULTS

### Characteristics of the patients and the tumors

**Figure 1:**
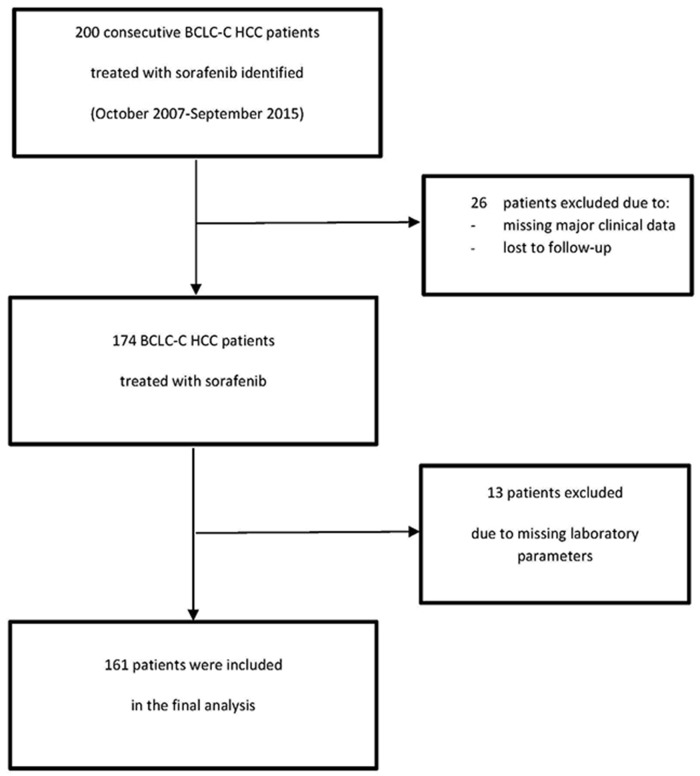
Flow chart of enrolled patients in this study

Out of 200 consecutive sorafenib-treated patients with advanced BCLC-C HCC meeting the inclusion criteria, 161 were included in the study (males: 87.0%; median (range) age: 67.1 (44.6-80.1); Eastern Cooperative Oncology Group performance status of 0-1: 87.0%) (Figure 1). The study population's baseline characteristics are summarized in Table 1. The median (range) follow-up period was 11.74 months (0.2–73.0) and the median duration of sorafenib therapy was 130 days. The median OS time was 9.1 months (7.9-10.1), and 148 patients (91.9%) died. The OS rates [95% CI] at 6, 12 and 24 months were 67.5% [59.7–74.2], 35.0% [27.6-42.4%] and 13.0% [8.1-19.0%], respectively. Chronic liver diseases (due to excessive alcohol consumption in 49.0% of the patients) were classified as CP-A and CP-B in 75.8% and 24.2% of cases, respectively. The cause of cirrhosis was multifactorial in 11.8% of the patients. Seven cases of HCC were diagnosed by liver biopsy; all the other were diagnosed using imaging. Extrahepatic spread was found in 62.7% of cases and vascular invasion was found in 44.7%. Prior to initiation of sorafenib, 95 patients (59.0%) had received one or more specific drugs. Sorafenib was withdrawn in 95.0% of the patients, following the onset of unacceptable toxicity (in 35.1% of withdrawals), tumor progression (21.4%), a worsening of liver function (28.6%) and death (14.9%). The patients’ baseline laboratory data are summarized in Table 2.

**Table 1:** Baseline demographic and clinical characteristics of patients with advanced HCC

Characteristic	Patients (n=161)
Mean age ±SD (years)	
Upon diagnosis of HCC	66.0 ±8.3
At initiation of sorafenib	67.2 ±8.6
Gender (n, %)	
Male	140 (86.96%)
Female	21 (13.04%)
BMI (kg/m^2^) (mean ± SD)	27.3 ±6.37^*^
PS (n, %)	
0	59 (36%)
1	81 (50.31%)
2	21 (13.04%)
Etiology of cirrhosis (n, %)	
Alcohol abuse	82 (50.93%)
Chronic hepatitis B	16 (9.94%)
Chronic hepatitis C	31 (19.25%)
Metabolic syndrome	31 (19.25%)
Hemochromatosis	7 (4.35%)
Others	8 (4.97%)
Cirrhosis (n, %)	140 (86.96%)
Child-Pugh stage (n, %)	
A5	79 (49.07%)
A6	43 (26.71%)
B7	21 (13.04%)
B8	13 (8.07%)
B9	5 (3.11%)
Ascites (n, %)	27 (16.77%)
Nodules (n, %)	
1-2	48 (29.81%)
≥3	113 (70.19%)
Macrovascular invasion (n, %)	72 (44.72%)
Lymph node involvement (n, %)	53 (32.92%)
Distant metastasis (n, %)	48 (29.81%)
Lung	24 (14.90%)
Bone	14 (8.70%)
Carcinomatosis	12 (7.45%)
Adrenal	6 (3.73%)
Others	2 (1.24%)
Previous treatments (n, %)	95 (59.01%)
Liver resection	17 (10.56%)
Liver transplantation	3 (1.86%)
TACE	67 (41.62%)
Radiofrequency ablation	15 (9.31%)
Stereotactic body radiation	6 (3.73%)
Intra-arterial iodine-131-iodized oil hepatic injection	16 (9.94%)

^*^missing data: n=3

Abbreviations: SD = standard deviation; BMI = body mass index; PS = performance status; MS= metabolic syndrome; TACE = transarterial chemoembolization;

**Table 2:** Baseline laboratory parameters in the study population (n=161)

Variable	Median; Mean (±SD)
Hemoglobin (g/L)	13.00; 12.98 ±1.99
WBC (×10^9^/L)	6.11; 6.60 ±2.94
Platelet count	163.00; 204.52 ±136.77
Neutrophil count (×10^9^/L)	4.27; 4.75 ±2.54
Lymphocyte count (×10^9^/L)	1.10; 1.21 ±0.59
Monocyte count (×10^9^/L)	0.49; 0.52 ±0.26
PT (%)	83.00; 81.63 ±12.86
Creatinine (μmol/L)	9.10; 11.35 ±11.78
MDRD (ml/min/1.73 m^2^)	88.00; 88.41 ±31.29
Total bilirubin (μmol/L)	17.00; 23.10 ±19.92
Albumin (g/dL)	35.90; 35.96 ±5.33
ALT (U/L)	48.00; 62.78 ±59.28
AST (U/L)	76.00; 105.02 ±110.66
AFP (ng/mL)	126.00; 13222.18 ±80027.31
GGT (U/L)	251.50; 334.94 ±285.26
ALP (U/L)	178.00; 236.38 ±238.28
NLR	3.97; 4.80 ±4.101
dNLR	2.28; 2.52 ±1.48
PLR	161.47; 200.90 ±181.269
LMR	2.28; 2.97 ±2.95
PNI	41.83; 42.03 ±26.550
SII (× 10^9^)	614.94; 1051.64 ±1317.67

Abbreviations: WBC= white blood cell count; PT= prothrombin time; ALT= alanine aminotransferase; AST = aspartate aminotransferase; AFp= alpha-fetoprotein; ALp= alkaline phosphatase; GGT= gamma-glutamyltranspeptidase; NLR= neutrophil-to-lymphocyte ratio; dNLR= derived neutrophil-to-lymphocyte ratio; PLR= platelet-to-lymphocyte ratio; LMR= lymphocyte-to-monocyte ratio; PNI= prognostic nutritional index; SII= systemic-immune inflammation index.

### Prognostic factors for HCC

The cut-off levels chosen for their clinical significance were the upper limits for WBC, PLT, ALT, AST, and ALP, and the lower limit for PT. The cut-offs for age, albumin and AFP were taken from literature reports of thresholds with proven prognostic value. The cut-offs for GGT, NLR, dNLR, PLR, LMR, PNI, and SII corresponded to the values that maximized the probability in the bivariate Cox model.

The results of the bivariate analysis are presented in Table 3. The variables significantly associated with OS were: BMI >25 kg/m2 (HR [95% CI]=0.63 [0.44-0.91]), macroscopic vascular invasion (HR [95% CI]=1.74 [1.26-2.42]), CP-B (HR [95% CI]=2.40 [1.75-3.47]), AST >38 IU/L (HR [95% CI]= 2.57 [1.63-4.03]), AFP ≥ 400 ng/mL (HR [95% CI]= 1.43 [1.03-2.00]), and ALP >120 IU/L (HR [95% CI]= 2.54 [1.64-3.95). The WBC and the platelet count alone were not predictive of OS, and there was no significant difference between treatment-naïve and pre-treated patients (HR [95% CI]=0.749 [0.54-1.04]; *p=*0.08), including those having undergone liver surgery. Each IBS was found to be a risk factor for OS, as follows: NLR ≥ 4 (HR [95% CI]= 1.74 [1.25-2.40]); dNLR ≥3 (HR [95% CI]= 2.28 [1.59-3.28]); PLR ≥ 200 (HR [95% CI]= 1.54 [1.09-2.18]); LMR <3 (HR [95% CI]= 1.45 [1.02-2.06]); PNI < 45 (HR [95% CI]= 2.01 [1.40-2.88]); and SII ≥600 × 10^9^ (HR [95% CI]= 1.54 [1.11-2.13]).

**Table 3:** Prognostic factors for overall survival in the study population: bivariate analyses

Variable	Description	HR [95% CI]	p value
Age ≥ 65	103(64.0%)	0.76 [0.55;1.07]	0.114
BMI ≥25 kg/m^2^	107(68.1%)	0.63 [0.44;0.91]	0.014
Ascites	27(16.77%)	0.83 [0.54;1.26]	0.380
Cirrhosis	140(87.0%)	1.22 [0.75;1.99]	0.412
Number of lesions≥3	113(70.2%)	1.30 [0.90;1.86]	0.151
Macroscopic vascular invasion	72(44.7%)	1.74 [1.26;2.42]	0.001
Lymph node involvement	53(32.9%)	1.36 [0.96;1.91]	0.080
Distant metastasis	48(24.2%)	0.90 [0.62;1.29]	0.550
Child Pugh score B *(vs A)*	39(24.2%)	2.40 [1.75;3.47]	<.001
Previous locoregional treatment	95(59.0%)	0.75 [0.54;1.04]	0.084
WBC >10 x109/L	14(8.70%)	1.56 [0.88;2.76]	0.130
Platelet count ≥150 G/L	88(55%)	0.98 [0.70;1.36]	0.882
PT < 70%	27(18%)	1.85 [1.20;2.84]	0.005
Total bilirubin >17 μmol/L	79(49.1%)	1.61 [1.16;2.22]	0.004
Albumin <3.5 g/dL	68(43.0%)	1.62 [1.16;2.26]	0.004
ALT >40 U/L	101(63.1%)	1.63 [1.16;2.30]	0.006
AST >38 U/L	133(82.6%)	2.57 [1.63;4.03]	<.001
AFP ≥400 ng/mL	60(37.3%)	1.43 [1.03;2.00]	0.033
ALP >120 U/L	129(81.1%)	2.54 [1.64;3.95]	<.001
GGT >250 U/L	81(51.9%)	1.67 [1.19;2.34]	.0027
NLR≥4	78(48.5%)	1.74 [1.25;2.40]	0.001
dNLR ≥3	45(28.1%)	2.28 [1.59;3.28]	<0.001
PLR≥200	54(33.8%)	1.54 [1.09;2.18]	0.014
LMR<3	112(69.6%)	1.45 [1.02;2.06]	0.037
PNI<45	106(67.1%)	2.01 [1.40;2.88]	<.001
SII≥600 (× 10^9^)	84(66.9%)	1.54 [1.11;2.13]	0.010

Abbreviations: WBC= white blood cell count; PT= prothrombin time; ALT= alanine aminotransferase; AST = aspartate aminotransferase; AFp= alpha-fetoprotein; ALp= alkaline phosphatase; GGT= gamma-glutamyltranspeptidase; NLR= neutrophil-to-lymphocyte ratio; dNLR= derived neutrophil-to-lymphocyte ratio; PLR= platelet-to-lymphocyte ratio; LMR= lymphocyte-to-monocyte ratio; PNI= prognostic nutritional index; SII = systemic-immune inflammation index

The WBC, PT, PLT, ALT, AST, ALP cut-offs were defined according to the clinical significance of their normal upper limit. The age, albumin and AFP cut-offs were chosen according to previous published thresholds with proven prognostic value. The GGT, NLR, dNLR, PLR, LMR, PNI, and SII cut-off levels corresponded to the value that maximized the probability in the bivariate Cox model.

**Figure 2:**
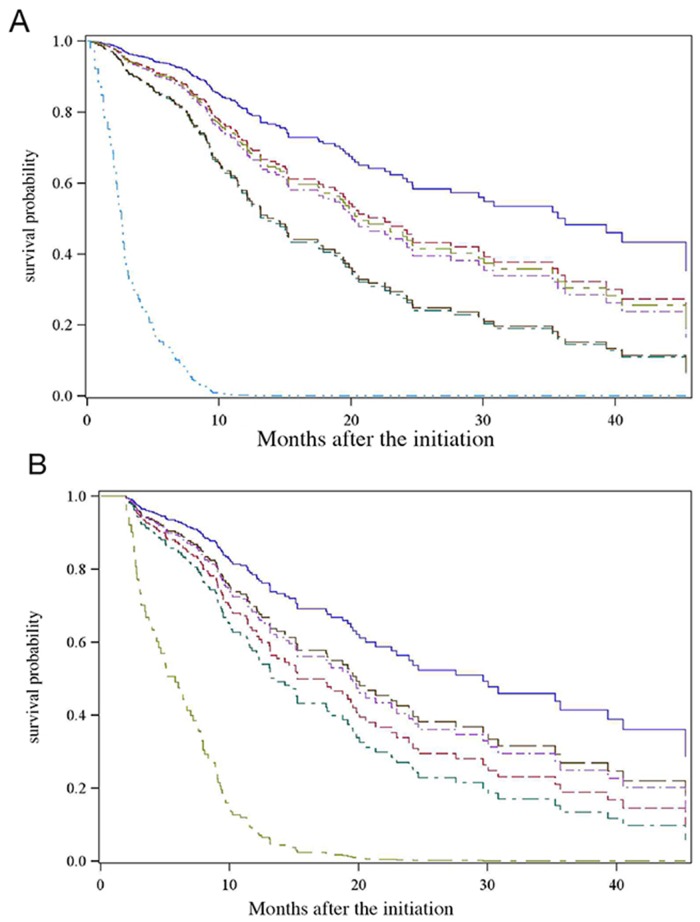
Kaplan-Meier estimates of survival curves with advanced BCLC-C HCC **(A)** Kaplan-Meier estimates of survival curves with advanced BCLC-C HCC, depending on the presence of the identified independent prognostic factors. (A) No independent risk factor. 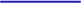; (B) BMI < 25 kg/m^2^. 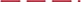; (C) Macroscopic vascular invasion 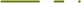.; (D) SII > 600.10^9^. 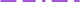; (E) Child-Pugh B 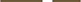; (F) AST > 38 UI/L. 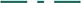; (G) Presence of all risk factors 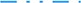. **(B)** Kaplan-Meier estimates of survival curves with advanced BCLC-C HCC in the subgroup of CP-A patients, depending on the presence of the identified independent prognostic factors. (A) no independent risk factor. 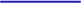; (B) BMI < 25 kg/m^2^. 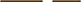; (C) macroscopic vascular invasion. 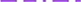; (D) SII > 600.10^9^. 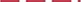; (E) AST > 38 UI/L. 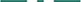; of all risk factors 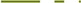.

The results of the multivariate analysis of the whole study population are summarized in Table 4. The final model identified five significant independent predictors of OS: AST >38 IU/L (HR 2.65 [1.67-4.19]); BMI < 25 kg/m^2^ (HR 1.55 [1.08-2.24]); SII >600 × 10^9^ (HR 1.72 [1.22-2.43]); CP-B (HR 2.59 [1.75-3.28]; and macroscopic vascular invasion (HR 1.63 [1.16-2.30]). The independent variables’ discriminant power for OS was represented with Kaplan-Meier survival curves (Figure 2A). The median survival time for patients (n=6) with all five independent risk factors was 2.6 months (versus 35.6 months for patients with none of the risk factors).

**Table 4:** Factors predicting overall survival (n=156): multivariate analyses

Variable	Multivariate analysis	
	HR [95% CI]	p value
BMI < 25 kg/m2	1.56 [1.08;2.24]	0.017
Macroscopic vascular invasion	1.74 [1.26;2.42]	0.001
Child Pugh score B *(vs. A)*	2.59 [1.75;3.83]	<.001
AST >38 IU/L	2.65 [1.67;4.19]	<.001
SII ≥600 (× 10^9^)	1.63 [1.16;4.19]	0.002

The multivariate model was also applied to a CP-A patient subgroup. SII ≥600 × 10^9^ was found to have negative prognostic value for OS (HR = 1.49 [1.01-2.20]), along with AST >38 IU/L (HR = 2.28 [1.37-3.79]), BMI < 25 kg/m^2^ (HR = 1.89 [1.25-2.86]) and macroscopic vascular invasion (HR = 1.57 [1.06-2.34]) (Table 5). The independent variables’ discriminant power for OS is shown in Figure 2B. Similarly, the median OS for CP-A patients (n=13) with all four independent risk factors was 5.8 months.

**Table 5:** Factors predicting overall survival in Child-Pugh A patients (n=119): multivariate analyses

Variable	Multivariate analysis	
	HR [95% CI]	p value
BMI < 25 kg/m2	1.89 [1.25;2.86]	0.003
Macroscopic vascular invasion	1.57 [1.06;2.34]	0.026
AST >38 IU/L	2.28 [1.37;3.79]	0.002
SII ≥600 (× 10^9^)	1.49 [1.06;2.20]	0.047

Abbreviations: BMI = body-mass index AST = aspartate aminotransferase; SII = systemic-immune inflammation index

## DISCUSSION

The present study assessed the prognostic value of six IBSs in advanced HCC patients subsequently treated with sorafenib. Our results demonstrate that IBSs are significantly correlated with OS, and that the SII is the strongest independent predictor of OS in sorafenib-treated patients with advanced HCC. The SII is a routine, reliable, simple, low-cost laboratory measurement. It is a more objective, comprehensive marker than indexes like the NLR and PLR or its components alone (i.e. neutrophil, lymphocyte and platelet counts). An elevated SII is suggestive of severe inflammatory status and/or a weak immune response. Given that sorafenib is not necessarily of benefit in CP-B patients [28], the SII is a valuable, independent predictive factor of OS when only CP-A patients are analyzed.

Our results are consistent with previous clinical studies in which a high SII was a risk factor for poor survival. The SII was initially described as an independent prognostic factor after curative resection for HCC BCLC 0+A in two independent Chinese cohorts, for both time to progression (HR [95% CI]=1.92 [1.04–3.54]; *p*=0.037) and OS (HR [95% CI]=2.10 [1.14–3.85]; *p=*0.017) [29]. The SII was also significantly associated with a poor OS following TACE in BCLC stage A, B and C HCC patients [27]. Advanced HCC patients receiving sorafenib with SII ≥ 360 showed also lower median PFS (2.6 vs. 3.9 months, *p*=0.026) and OS (5.6 vs. 13.9 months, *p*=0.027) compared to those with SII < 360 [30]. As in the present study, the SII was a better prognostic factor than the other indices (PLR, NLR, tumor number, AFP, etc.). Hu and al. concluded that the high recurrence rate in resected patients with high SII scores might be due to high circulating tumor cell counts. Hu and al.'s optimal SII cut-off (330 × 10^9^) was lower than the value determined in the present study (600 × 10^9^), but most of the Chinese patients were hepatitis-B-virus–positive and staged as BCLC 0 or A. Our findings agree with those of another study in which patients with BCLC stage C HCC also had significantly higher SII scores than those with BCLC stages A to B HCC (*p<* 0.0001) [27] and with those showing SII as an independent prognostic factor for OS in BCLC-C HCC patients [30]. Moreover, our cut-off is similar to the value selected in patients with metastatic renal carcinoma or metastatic colorectal cancer [31, 32].

A systemic inflammatory response is known to promote tumor angiogenesis, invasion and metastasis through a variety of mechanisms, and is associated with poor survival in various types of cancer [33]. Firstly, lymphocytes enhance the antitumor immune response and control tumor defense by inducing cytotoxic cell death and inhibiting tumor cell proliferation and migration. Secondly, neutrophils promote cancer cell invasion, proliferation and metastasis, and modify the tumor micro-environment [34, 35]. Thirdly, platelets interact with tumor cells and facilitate tumor cell survival and metastasis through a variety of mechanisms, including the protection of circulating tumor cells against shear stresses [18,36–40].

The other independent prognostic factors for poor OS identified in our study have often been described in the literature; they include an elevated AST, CP-B status, macroscopic vascular invasion [8, 28, 41, 42], and BMI < 25 kg/m^2^ [43-45] (although BMI has not been analyzed very frequently in the literature). Low BMI (< 25 kg/m^2^) is associated with dose-limiting toxicity of sorafenib in patients with advanced HCC, and especially in patients with sarcopenia [43-45].

We found that the median OS for patients with all of the risk factors was 2.6 months (versus 5.8 months for a CP-A patient). Thus, a physician may decide not to initiate sorafenib treatment in patients meeting most of the criteria.

The present study had several limitations. Its retrospective design meant that CRP was not assayed systematically; hence, we could not assess the Glasgow prognostic score, the modified Glasgow prognostic score or the CRP/albumin ratio [46-48]. Furthermore, this was a single-center study. Lastly, the number of patients with missing data (n=26 patients) accounted for 13% of the overall study population.

In conclusion, an abnormally high AST level, SII >600 × 10^9^, BMI < 25 kg/m^2^, macrovascular invasion, and CP-B cirrhosis are predictive of poor OS in patients with advanced HCC. The SII is a non-invasive, routine, low cost index with prognostic value in patients with advanced HCC. It can potentially be used to guide the treatment strategy. Confirmation of the present results in prospective studies is now required.

## MATERIALS AND METHODS

### Patients

Eligible patients were treated with sorafenib for an indication of advanced HCC (stage C, according to the updated BCLC staging system [5]) in the Department of Hepatology and Gastroenterology at Nancy University Medical Center (Nancy, France) from October 2007 to September 2015. The radiologic and/or histologic diagnostic criteria for HCC were based on the American Association for the Study of the Liver guidelines [6]. Some patients had undergone liver transplantation, surgical resection and/or locoregional therapies (radiofrequency ablation, transarterial chemoembolization (TACE), radio-embolization with microspheres containing yttrium-90, or stereotaxic body radiation therapy) prior to the initiation of sorafenib treatment sorafenib. All patients had undergone contrast-enhanced computed tomography or magnetic resonance imaging prior to the initiation of sorafenib treatment. The study was approved by the local institutional review board (Comité de réflexion éthique nancéien hospitalo-universitaire CREHNU).

### Data collection

The Department of Hepatology and Gastroenterology's case database for the period between October 2007 and September 2015 was systematically searched with the term “sorafenib”. All patients involved in clinical trials were included, after study blinding had been lifted. The patients’ demographic, clinical and biological data were collected retrospectively from their medical charts. Variables recorded at inclusion were as follows: gender, age, body mass index (BMI), Child Pugh (CP) score (albumin, prothrombin time (PT), total bilirubin, ascites, and encephalopathy), the etiology of cirrhosis (hepatitis C or B virus, alcoholic or non-alcoholic steatohepatitis, or hemochromatosis), the number of tumors and the tumor characteristics. The results of routine blood tests, liver function tests and alpha-fetoprotein (AFP) assays performed in the month preceding the initiation of sorafenib treatment were also recorded, if available.

To identify baseline risk factors associated with OS in HCC, we evaluated 10 clinical/radiologic parameters (age, BMI, ascites, cirrhosis, number of lesion >3, macroscopic vascular invasion, lymph node or distant metastasis, the CP score, and previous locoregional treatment), 11 biological factors (the white blood count (WBC), the platelet count, the PT, and total bilirubin, albumin, ALT, AST, AFP, and alkaline phosphatase (ALP) levels), and six IBSs (the neutrophil-to lymphocyte ratio (NLR), the derived NLR, the platelet-to-lymphocyte ratio (PLR), the monocyte-to-lymphocyte ratio (MLR), the prognostic nutritional index (PNI) and the systemic-immune inflammation index (SII) [29]. The six IBSs at baseline were calculated from serum complete blood counts using the following equations, where M, L, N, P, W are the absolute monocyte, lymphocyte, neutrophil, platelet and white blood-cell counts, respectively: NLR = N/L; dNLR = N/(W-N); MLR = M/L; PLR = P/L; PNI = serum albumin + 0.005 x L; SII = P × N/L.

### Treatments and patient follow-up

The prescribed dose of sorafenib was 400 mg *per os* *bid*. The patients’ clinical and toxicity profiles were assessed at least every 4 weeks. If adverse drug reactions occurred (and depending on the latter's grade), the dose of sorafenib was reduced. Treatment was continued until the onset of unacceptable toxicity, radiologic or clinical progression, death or patient refusal.

### Statistical analysis

Statistical analysis was performed using SAS software (SAS Institute Inc., Cary, NC). The threshold for statistical significance was set to p<0.05. OS was defined as the time interval between the first day of sorafenib treatment and the day of death. Surviving patients were censored at the date of last follow-up or 4 years after the start of sorafenib treatment. The association between OS and each prognostic factor was first investigated using bivariate and multivariate Cox proportional hazards models. The results were expressed as a hazard ratio (HR) [95% confidence interval (CI)]. Quantitative variables were transformed into binary variables. We used two approaches to choose thresholds: (i) assessment of known clinical significance; (2) value that best separates patient outcomes according the minimum P-value method in the bivariate Cox model. The validity of the proportional hazard (PH) assumption was checked by determining the scaled Schoenfeld residuals (SSR). The PH assumption was tested for each covariate by correlating the corresponding SSR with the rank of time [49]. All variables with a p-value <0.1 in the bivariate Cox model were introduced in a multivariate Cox model with backward selection at p=0.10. The model's stability was investigated using a bootstrap resampling method [50]. A multivariate Cox model with backward selection (p<0.1) was run on each of these replicates. The variable was retained in the final model if it was selected in at least 70% of the 500 analyses [50]. To address the problem of correlated variables, the selection frequencies of all possible pairs of variables were also considered. If a pair of variables was selected in more than 90% of replicates, the covariate with the higher inclusion frequency was selected [50]. The results of the final multivariate model were presented as the adjusted HR [95% CI]. The same model was applied to a CP-A subgroup. For each independent prognostic factor, the discriminant power for OS in a multivariate analysis was represented as a Kaplan-Meier survival curve. The predicted survival function (S(t)) for particular sets of risk factors were computed using the “phreg” procedure and the “baseline” statement in SAS. The median survival time was determined as the smallest value of the time t so that S(t)≤0.50.

## References

[1] Ferlay J, Soerjomataram I, Dikshit R, Eser S, Mathers C, Rebelo M, Parkin DM, Forman D, Bray F (2015). Cancer incidence and mortality worldwide: Sources, methods and major patterns in GLOBOCAN 2012. Int J Cancer.

[2] Park JW, Chen M, Colombo M, Roberts LR, Schwartz M, Chen PJ, Kudo M, Johnson P, Wagner S, Orsini LS, Sherman M (2015). Global patterns of hepatocellular carcinoma management from diagnosis to death: the BRIDGE Study. Liver Int.

[3] Llovet JM, Ricci S, Mazzaferro V, Hilgard P, Gane E, Blanc JF, de Oliveira AC, Santoro A, Raoul JL, Forner A, Schwartz M, Porta C, Zeuzem S (2008). Sorafenib in advanced hepatocellular carcinoma. N Engl J Med.

[4] Cheng AL, Kang YK, Chen Z, Tsao CJ, Qin S, Kim JS, Luo R, Feng J, Ye S, Yang TS, Xu J, Sun Y, Liang H (2009). Efficacy and safety of sorafenib in patients in the Asia-Pacific region with advanced hepatocellular carcinoma: a phase III randomised, double-blind, placebo-controlled trial. Lancet Oncol.

[5] Llovet JM, Brú C, Bruix J (1999). Prognosis of hepatocellular carcinoma: the BCLC staging classification. Semin Liver Dis.

[6] Bruix J, Sherman M, American Association for the Study of Liver Diseases (2011). Management of hepatocellular carcinoma: an update. Hepatol Baltim Md.

[7] European Association For The Study Of The Liver, European Organisation For Research And Treatment Of Cancer (2012). EASL-EORTC clinical practice guidelines: management of hepatocellular carcinoma. J Hepatol.

[8] Llovet JM, Peña CEA, Lathia CD, Shan M, Meinhardt G, Bruix J (2012). Plasma Biomarkers as Predictors of Outcome in Patients with Advanced Hepatocellular Carcinoma. Clin Cancer Res.

[9] McMillan DC (2009). Systemic inflammation, nutritional status and survival in patients with cancer. Curr Opin Clin Nutr Metab Care.

[10] Proctor MJ, Morrison DS, Talwar D, Balmer SM, Fletcher CD, O’Reilly DS, Foulis AK, Horgan PG, McMillan DC (1990). A comparison of inflammation-based prognostic scores in patients with cancer A Glasgow Inflammation Outcome Study. Eur J Cancer Oxf Engl.

[11] Shrotriya S, Walsh D, Bennani-Baiti N, Thomas S, Lorton C (2015). C-Reactive Protein Is an Important Biomarker for Prognosis Tumor Recurrence and Treatment Response in Adult Solid Tumors: A Systematic Review. PloS One.

[12] Petrelli F, Cabiddu M, Coinu A, Borgonovo K, Ghilardi M, Lonati V, Barni S (2015). Prognostic role of lactate dehydrogenase in solid tumors: a systematic review and meta-analysis of 76 studies. Acta Oncol Stockh Swed.

[13] Templeton AJ, McNamara MG, Šeruga B, Vera-Badillo FE, Aneja P, Ocaña A, Leibowitz-Amit R, Sonpavde G, Knox JJ, Tran B, Tannock IF, Amir E (2014). Prognostic role of neutrophil-to-lymphocyte ratio in solid tumors: a systematic review and meta-analysis. J Natl Cancer Inst.

[14] Templeton AJ, Ace O, McNamara MG, Al-Mubarak M, Vera-Badillo FE, Hermanns T, Seruga B, Ocaña A, Tannock IF, Amir E (2014). Prognostic role of platelet to lymphocyte ratio in solid tumors: a systematic review and meta-analysis. Cancer Epidemiol Biomark Prev.

[15] Roxburgh CS, McMillan DC (2010). Role of systemic inflammatory response in predicting survival in patients with primary operable cancer. Future Oncol Lond Engl.

[16] Bugada D, Allegri M, Lavand’homme P, De Kock M, Fanelli G (2014). Inflammation-based scores: a new method for patient-targeted strategies and improved perioperative outcome in cancer patients. BioMed Res Int.

[17] Nishijima TF, Muss HB, Shachar SS, Tamura K, Takamatsu Y (2015). Prognostic value of lymphocyte-to-monocyte ratio in patients with solid tumors: A systematic review and meta-analysis. Cancer Treat Rev.

[18] Pang Q, Zhang JY, Xu XS, Song SD, Qu K, Chen W, Zhou YY, Miao RC, Liu SS, Dong YF, Liu C (2015). Significance of platelet count and platelet-based models for hepatocellular carcinoma recurrence. World J Gastroenterol.

[19] Chan AWH, Chan SL, Wong GLH, Wong VWS, Chong CCN, Lai PBS, Chan HLY, To KF (2015). Prognostic Nutritional Index (PNI) Predicts Tumor Recurrence of Very Early/Early Stage Hepatocellular Carcinoma After Surgical Resection. Ann Surg Oncol.

[20] Liu Y, Wang ZX, Cao Y, Zhang G, Chen WB, Jiang CP (2016). Preoperative inflammation-based markers predict early and late recurrence of hepatocellular carcinoma after curative hepatectomy. Hepatobiliary Pancreat Dis Int HBPD INT.

[21] Li X, Chen ZH, Xing YF, Wang TT, Wu DH, Wen JY, Chen J, Lin Q, Dong M, Wei L, Ruan DY, Lin ZX, Wu XY (2015). Platelet-to-lymphocyte ratio acts as a prognostic factor for patients with advanced hepatocellular carcinoma. Tumour Biol J.

[22] Qi X, Li J, Deng H, Li H, Su C, Guo X (2016). Neutrophil-to-lymphocyte ratio for the prognostic assessment of hepatocellular carcinoma: A systematic review and meta-analysis of observational studies. Oncotarget.

[23] Lai Q, Castro Santa E, Rico Juri JM, Pinheiro RS, Lerut J (2014). Neutrophil and platelet-to-lymphocyte ratio as new predictors of dropout and recurrence after liver transplantation for hepatocellular cancer. Transpl Int.

[24] Lin ZX, Ruan DY, Li Y, Wu DH, Ma XK, Chen J, Chen ZH, Li X, Wang TT, Lin Q, Wen JY, Wu XY (2015). Lymphocyte-to-monocyte ratio predicts survival of patients with hepatocellular carcinoma after curative resection. World J Gastroenterol.

[25] Wu SJ, Lin YX, Ye H, Li FY, Xiong XZ, Cheng NS (2016). Lymphocyte to monocyte ratio and prognostic nutritional index predict survival outcomes of hepatitis B virus-associated hepatocellular carcinoma patients after curative hepatectomy. J Surg Oncol.

[26] Yamamura K, Sugimoto H, Kanda M, Yamada S, Nomoto S, Nakayama G, Fujii T, Koike M, Fujiwara M, Kodera Y (2014). Comparison of inflammation-based prognostic scores as predictors of tumor recurrence in patients with hepatocellular carcinoma after curative resection. J Hepato-Biliary-Pancreat Sci.

[27] Yang Z, Zhang J, Lu Y, Xu Q, Tang B, Wang Q, Zhang W, Chen S, Lu L, Chen X (2015). Aspartate aminotransferase-lymphocyte ratio index and systemic immune-inflammation index predict overall survival in HBV-related hepatocellular carcinoma patients after transcatheter arterial chemoembolization. Oncotarget.

[28] Marrero JA, Kudo M, Venook AP, Ye SL, Bronowicki JP, Chen XP, Dagher L, Furuse J, Geschwind JFH, de Guevara LL, Papandreou C, Takayama T, Sanyal AJ (2016). Observational registry of sorafenib use in clinical practice across Child-Pugh subgroups: the GIDEON study. J Hepatol.

[29] Hu B, Yang XR, Xu Y, Sun YF, Sun C, Guo W, Zhang X, Wang WM, Qiu SJ, Zhou J, Fan J (2014). Systemic immune-inflammation index predicts prognosis of patients after curative resection for hepatocellular carcinoma. Clin Cancer Res.

[30] Casadei Gardini A, Scarpi E, Faloppi L, Scartozzi M, Silvestris N, Santini D, de Stefano G, Marisi G, Negri FV, Foschi FG, Valgiusti M, Ercolani G, Frassineti GL (2016). Immune inflammation indicators and implication for immune modulation strategies in advanced hepatocellular carcinoma patients receiving sorafenib. Oncotarget.

[31] Lolli C, Basso U, Derosa L, Scarpi E, Sava T, Santoni M, Crabb SJ, Massari F, Aieta M, Conteduca V, Maruzzo M, La Russa F, Wheater M (2016). Systemic immune-inflammation index predicts the clinical outcome in patients with metastatic renal cell cancer treated with sunitinib. Oncotarget.

[32] Passardi A, Scarpi E, Cavanna L, Dall’Agata M, Tassinari D, Leo S, Bernardini I, Gelsomino F, Tamberi S, Brandes AA, Tenti E, Vespignani R, Frassineti GL (2016). Inflammatory indexes as predictors of prognosis and bevacizumab efficacy in patients with metastatic colorectal cancer. Oncotarget.

[33] Diakos CI, Charles KA, McMillan DC, Clarke SJ (2014). Cancer-related inflammation and treatment effectiveness. Lancet Oncol.

[34] Mantovani A, Allavena P, Sica A, Balkwill F (2008). Cancer-related inflammation. Nature.

[35] Hanahan D, Weinberg RA (2011). Hallmarks of cancer: the next generation. Cell.

[36] Labelle M, Begum S, Hynes RO (2014). Platelets guide the formation of early metastatic niches. Proc Natl Acad Sci U S A.

[37] Labelle M, Begum S, Hynes RO (2011). Direct signaling between platelets and cancer cells induces an epithelial-mesenchymal-like transition and promotes metastasis. Cancer Cell.

[38] Schumacher D, Strilic B, Sivaraj KK, Wettschureck N, Offermanns S (2013). Platelet-derived nucleotides promote tumor-cell transendothelial migration and metastasis via P2Y2 receptor. Cancer Cell.

[39] Bihari C, Rastogi A, Shasthry SM, Bajpai M, Bhadoria AS, Rajesh S, Mukund A, Kumar A, Sarin SK (2016). Platelets contribute to growth and metastasis in hepatocellular carcinoma. APMIS Acta Pathol Microbiol Immunol Scand.

[40] Xue TC, Ge NL, Xu X, Le F Zhang BH, Wang YH (2016). High platelet counts increase metastatic risk in huge hepatocellular carcinoma undergoing transarterial chemoembolization. Hepatol Res.

[41] Hollebecque A, Cattan S, Romano O, Sergent G, Mourad A, Louvet A, Dharancy S, Boleslawski E, Truant S, Pruvot FR, Hebbar M, Ernst O, Mathurin P (2011). Safety and efficacy of sorafenib in hepatocellular carcinoma: the impact of the Child-Pugh score. Aliment Pharmacol Ther.

[42] Lee S, Kim BK, Kim SU, Park SY, Kim JK, Lee HW, Park JY, Kim DY, Ahn SH, Tak WY, Kweon YO, Lee JI, Lee KS (2014). Clinical outcomes and prognostic factors of patients with advanced hepatocellular carcinoma treated with sorafenib as first-line therapy: a Korean multicenter study. J Gastroenterol Hepatol.

[43] Hiraoka A, Hirooka M, Koizumi Y, Izumoto H, Ueki H, Kaneto M, Kitahata S, Aibiki T, Tomida H, Miyamoto Y, Yamago H, Suga Y, Iwasaki R (2016). Muscle volume loss as a prognostic marker in hepatocellular carcinoma patients treated with sorafenib. Hepatol Res.

[44] Huillard O, Mir O, Peyromaure M, Tlemsani C, Giroux J, Boudou-Rouquette P, Ropert S, Delongchamps NB, Zerbib M, Goldwasser F (2013). Sarcopenia and body mass index predict sunitinib-induced early dose-limiting toxicities in renal cancer patients. Br J Cancer.

[45] Mir O, Coriat R, Blanchet B, Durand JP, Boudou-Rouquette P, Michels J, Ropert S, Vidal M, Pol S, Chaussade S, Goldwasser F (2012). Sarcopenia predicts early dose-limiting toxicities and pharmacokinetics of sorafenib in patients with hepatocellular carcinoma. PloS One.

[46] Li M, Bi X, Li Z, Huang Z, Han Y, Zhou J, Zhao J, Zhang Y, Zhao H, Cai J (2015). Prognostic Role of Glasgow Prognostic Score in Patients With Hepatocellular Carcinoma: A Systematic Review and Meta-Analysis. Medicine (Baltimore).

[47] Ni XC, Yi Y, Fu YP, He HW, Cai XY, Wang JX, Zhou J, Cheng YF, Jin JJ, Fan J, Qiu SJ (2015). Prognostic Value of the Modified Glasgow Prognostic Score in Patients Undergoing Radical Surgery for Hepatocellular Carcinoma. Medicine (Baltimore).

[48] Kinoshita A, Onoda H, Imai N, Iwaku A, Oishi M, Tanaka K, Fushiya N, Koike K, Nishino H, Matsushima M (2014). The C-Reactive Protein/Albumin Ratio, a Novel Inflammation-Based Prognostic Score, Predicts Outcomes in Patients with Hepatocellular Carcinoma. Ann Surg Oncol.

[49] Therneau TM (1996). Extending the Cox model.

[50] Sauerbrei W (1999). The use of resampling methods to simplify regression models in medical statistics. Appl Stat.

